# Optimizing Extraction of Bioactive Compounds from Grape Pomace to Produce an Eco-Friendly Smart Straw

**DOI:** 10.1021/acsomega.6c05389

**Published:** 2026-10-15

**Authors:** Luis Fernando Zitei-Baptista, Maisa Mitiko Takahashi, Ivone Yanira Choquetico Iquiapaza, Delia Rita Tapia-Blácido

**Affiliations:** Department of Chemistry, 124588 Faculty of Philosophy, Sciences and Letters of Ribeirão Preto, Ribeirão Preto, São Paulo 14040-901, Brasil

## Abstract

Grape pomace (GP) is a byproduct rich in bioactive compounds. This study optimized the extraction of these compounds using a Central Composite Rotational Design (CCRD 2^2^), evaluating temperature (41–69 °C) and time (0.8–3.2 h). The optimized extract (GPE) showed high phenolic content (163.99 mg GAE/L), anthocyanins (136.46 mg C3GE/L), and antioxidant activity (69.21% DPPH). GPE was incorporated into babassu mesocarp starch (BS) films, producing an active smart film (GPE-BS) that changed color at extreme pH (2 and 13). The GPE-BS film also showed improved hydrophobicity, antioxidant activity, and elongation at break compared to BS films (control). When tested as a straw for sugar-free cola, the film maintained structural integrity for 2 h. Thus, GPE-BS films are promising for smart food packaging or as edible straws.

## Graphical abstract

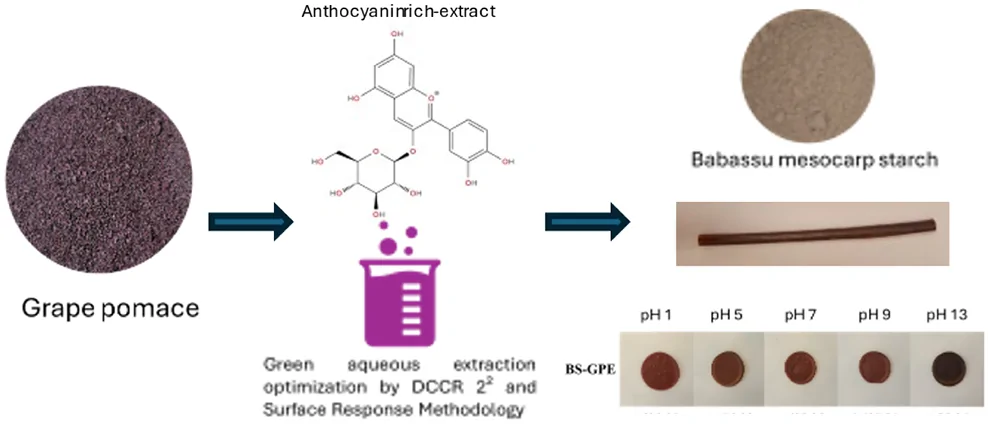

## 1 Introduction

Searching for new eco-materials to replace synthetic plastics is paramount because single-use plastics, such as packaging and straws, act as intermediates for microplastics to migrate to consumers. Single-use plastics have been banned by the European Union, which contrasts with their current use in the United States.^1,2^ Single-use plastics can release particles or fibers with sizes ranging from 0.11 to 8000 μm, at concentrations ranging from 1.13 to 38.0 mg/item or over 1.6 × 10^7^ particles/L for liquids.^3–5^ Microplastic migration causes microplastics to accumulate in the human body, which leads to respiratory, gastrointestinal, and neurological issues.^6,7^ Environmentally friendly alternatives, e.g., natural polymers such as polysaccharides and proteins, have been studied.^8,9^ These natural polymers can be found in agro-industrial waste, which makes this waste a sustainable alternative for obtaining environmentally friendly materials.^10,11^

Brazil is a major producer of grapes: this country produced around 1.7 million tons of grapes in 2021.^12^ Approximately 75% of harvested grapes is intended for producing wine, and 20–30% of this amount represents waste products, the so-called grape pomace, which consists of skin, pulp, seeds, and stalks.^13–15^ In 2022, the Brazilian southern region produced around 446 million liters of wine.^16^ The grape residue resulting from the wine industry is generally employed to obtain animal feed.^17^

Grape pomace (GP) is an important source of bioactive compounds, such as phenolic acids (caffeic, gallic, protocatechuic, 4-hydroxybenzoic, and syringic acids), phenolic alcohols (hydroxytyrosol), flavonoids (catechin, epicatechin, quercetin-3-O-rhamnoside, and luteolin), stilbenes (resveratrol), and proanthocyanidins.^18,19^ Some bioactive compounds exhibit excellent biological activities, e.g., anticancer, anti-inflammatory, and antihypertensive action.^20^ Among flavonoids, flavonols and anthocyanins are the primary phenolic compounds in grapes.^21,22^ These compounds have interesting bioactive properties that benefit human health.^23–26^ Anthocyanins are soluble in water and can be extracted with this solvent, thereby avoiding the use of organic solvents. In addition, anthocyanins change color when the pH is altered; therefore, they could be employed to produce smart edible films. Furthermore, anthocyanins can substitute for plasticizers in film formulations without impairing the final integrity of the film. Smart films were obtained by adding various biopolymeric matrices, such as chitosan, gelatin, cellulose, and starch, to grape pomace extract. These materials have been used as coatings for food items, such as fruit and fish.^80^ However, no studies were found on smart films using babassu mesocarp starch and grape pomace extracts. In addition, few studies in the literature focus on the development of straws.^81^

Babassu, a coconut of the *Arecaceae* family, grows on palm trees in northeastern Brazil. The fruit consists of the epicarp (fibrous husk), the mesocarp (representing about 23% of the fruit), and the endocarp (containing the babassu kernels).^79^ Babassu oil, which is used in the food, cosmetic, and pharmaceutical industries, is extracted from the kernels.^27^ In this context, the babassu mesocarp could be considered a byproduct of babassu oil production. Thanks to its high starch content (around 80%) and its phenolic compounds, it has attracted the attention of researchers as a potential source of new biobased materials.^78^ The presence of phenolic compounds in the babassu mesocarp starch results in films that exhibit antioxidant properties. Therefore, they have the potential to be used for active packaging, which is one of the main areas of focus for our research group.^28–32^ However, these films do not have smart properties. In this study, we therefore considered using grape pomace extract (GPE) to produce smart films from babassu mesocarp starch, building on our previous research on this raw material. Smart films are sensitive to changes in temperature and pH levels, making them ideal for preserving food and controlling its quality during storage.^33,34^

The aims of this study are (1) to optimize extraction of bioactive compounds from GP using a solid–liquid extraction process that is easily reproducible on an industrial scale. The optimization was performed using Central Composite Design (CCD) and Response Surface Methodology (RSM), and (2) to produce an active smart film based on babassu mesocarp starch, with the addition of GPE, for use as a straw for cola soda. In addition, we pretend to add value to a raw material derived from Brazil’s socio-biodiversity and to propose a new approach for the production of plastic-free smart straws.

## 2 Experimental Section

### 2.1 Materials

Grape pomace (GP) containing skin and seeds was provided by Terras Altas winery, located in Ribeirão Preto, SP, Brazil. Grapes were planted in August 2017 and harvested on 13 July 2022. Grapes of the Syrah variety (*Vitis vinifera*), grafted onto IAC766 and Paulsen rootstocks, were used. GP was dried at 50 °C for 24 h, ground, and sieved through a 45-mesh sieve.

Babassu was harvested in January of 2022. The babassu mesocarp flour, a byproduct of babassu oil production, was provided by Florestas Brasileiras S.A.

### 2.2 Extraction of Babassu Mesocarp Starch

Starch was isolated from babassu mesocarp by wet milling with distilled water (neutral pH), as described by Maniglia and Tapia-Blácido,^35^ with some adaptations. The babassu mesocarp flour was macerated with water at a 2:1 water/babassu flour ratio and 15 °C for 24 h. The flour suspension was ground in an Oster Versatile 1.5-L blender (model 006844-017-000) operating at maximum power for 3 min and sieved through 80-, 200-, and 270-mesh sieves. This process was repeated four times. After sieving, the suspension that passed through the sieves was left to rest in the fridge for starch to separate. The supernatant was discarded, and starch was heated at 35 °C in a forced circulation oven (Quimis, Q314 M model, Brazil) for 24 h to give the so-called babassu mesocarp starch (BS).

### 2.3 Grape Pomace and Babassu Mesocarp Starch Characterization

The GP and BS centesimal compositions, particularly the moisture (012/IV), ash (018/IV), and protein (037/IV) contents, were analyzed by using the methods described by Instituto Adolfo Lutz.^36^ The lipid content was analyzed by employing the method proposed by Bligh and Dyer.^37^ The carbohydrate content was calculated by difference, according to eq 1.

1 Carbohydrates⁡(%)=100−[Moisture⁡(%)+Ash⁡(%)+Protein⁡(%)+Lipids⁡(%)]

The amylose content was determined by the iodine colorimetric method, as described by Juliano et al.^38^

The size of the GP fractions obtained during the sieving procedure, namely, fraction M and fraction P, which passed and was retained in the 45-mesh sieve, respectively, and fraction G (nonmilled GP), was measured on a Beckman Coulter LS 13 320 Particle Size Analyzer (Beckman Coulter, USA) coupled to the Tornado module (Dry Powder Module).

### 2.4 Effect of Solvent Type and Grape Pomace Granulometry on the Extraction of Bioactive Compounds

Initially, several preliminary tests were conducted to determine the best solvent and particle size of the GP for the extraction of bioactive compounds from the GP. Four types of solvents were tested: water (H_2_O), ethanol (Et), acidic water (H_2_OAc), and acidic ethanol (EtAc),^82^ which was used as the primary reference in the literature for comparison with the other methods studied here. The GP fraction with medium particle size (Fraction M) was used in this stage. After selecting the solvent type, extractions were performed on the three GP fractions (P, M, and G) to determine the optimal particle size for the extraction of bioactive compounds. The total phenolic content (TPC), total anthocyanin content (TAC), antioxidant activity (AOA), and soluble solids content of the GP extracts were determined. All of these preliminary extractions were performed under the following conditions: 10 g of GP was used in 100 mL of solvent, and extraction was carried out at 28 °C for 2 h.

### 2.5 Optimizing Extraction of Bioactive Compounds by Using Central Composite Rotatable Design and Response Surface Methodology

Once the better green solvent and granulometry of grape pomace had been determined, a Central Composite Rotatable Design (CCRD) and Response Surface Methodology (RSM) were employed to determine the optimal conditions for extracting a high-TPC and TAC product. The two factors studied were temperature (X_1_), which ranged from 41 to 69 °C, and time (X_2_), which ranged from 0.8 to 3.2 h. These factors were chosen based on preliminary tests conducted using a fractional DoE of 2^4–1^ (results not shown), in which the main effects of mass percentage, time, temperature, and pH were studied. The factors that had the greatest statistical influence on the content of bioactive compounds in GPE were temperature and time. Studying these factors on an industrial scale would allow the results of this article to be easily transferred within the academia-industry interface. The responses were the total phenolic content (TPC), the total anthocyanin content (TAC), and the antioxidant activity (AOA).

CCRD allows a second-order polynomial regression equation to be obtained for each analyzed response (eq 2). This makes it possible to visualize the relationship between independent variables (factors) and dependent variables (responses).^39,40^

2 $$y=\beta_{o}+\sum \beta_{i}x_{i}+\sum \beta_{ii}x_{i}^{2}+\sum \beta_{ij}x_{i}x_{j}$$

where *y* is the predicted mean response, β_*o*_ is the constant term of the model, β_*i*_ are the coefficients of the linear terms, β_*ii*_ are the coefficients of the quadratic terms, and β_*ij*_ are the coefficients of the interaction terms.

In a CCD, each independent variable (factor) has five levels: −α, −1, 0, +1, and +α. Axial points (±α) are added to estimate the quadratic effects.^41^ Specifically, this study considered two independent variables for optimization: temperature (X_1_), which ranged from 41 to 69 °C; and time (X_2_), which ranged from 0.8 to 3.2 h. Table 3 presents the CCRD 2^2^ matrix containing the X_1_ and X_2_ combinations for each experiment with the corresponding coded and real values. The experiments were performed in duplicate. The dependent variables (responses) were TPC, TAC, and AOA.

The obtained data were statistically analyzed using Statistica 14.0 software. How the factors affected the responses was evaluated, and the regression coefficients were analyzed so that fitted mathematical models could be achieved. An analysis of variance (ANOVA) and an F-test were performed to evaluate the statistical significance and prediction of the fitted mathematical models. If F_regression_ ≫ F_tab_ (*p*-value < 0.05), the regression mathematical model was considered significant, and if F_lack of fit_ < F_tab_ (*p*-value > 0.05), the regression mathematical model was considered predictive.^39^ If these two conditions were satisfied, the model could be used to model the response surface.

The second-order mathematical models that the F-test showed to be significant and predictive were used to determine the optimal extraction conditions. For this purpose, desirability function methodology was employed to optimize multiple responses. This methodology involves transforming each estimated response (y_i_) into a desirability function (d­(yi)), which ranges from 0 (undesirable response) to 1 (the most desirable response). Then, an overall desirability function (D) is calculated as a geometric average of all of the individual desirability functions (d).^42^

### 2.6 Grape Pomace Extract Characterization

The TPC, TAC, AOA, and soluble solid contents of GPE were determined. The GPE TAC was determined by using the monomeric anthocyanin assay proposed by Lee et al.^43^ This involved measuring the GPE absorbance at 520 and 700 nm on a spectrophotometer (Femto 600 Plus, Brazil) at different pH values (1 and 4.5). TAC is expressed as milligrams of cyanidin-3-glucoside equivalent per liter of GPE (mg of cya-3-glu equivalent/L).

The GPE TPC was determined by using the methodology proposed by Singleton and Rossi^44^ on the basis of the Folin-Ciocalteu reagent. Specifically, the sample was prepared by using 0.25 mL of GPE and 2.75 mL of 3% Folin-Ciocalteu reagent. Then, 0.25 mL of 10% sodium carbonate was added, and the reaction mixture was allowed to proceed for 1 h. Absorbance at 765 nm was measured on a spectrophotometer (Femto 600 Plus, Brazil). TPC is expressed as milligrams of gallic acid equivalent per liter of GPE (milligrams of GAE/L). TPC was obtained by using a calibration curve for gallic acid concentrations ranging from 0 to 100 mg/L (absorbance = 0.0069TPC + 0.0134, R^2^ = 0.998).

The GPE AOA was analyzed by using the DPPH assay.^45^ A DPPH (2,2-diphenyl-1-picrylhydrazyl) solution was prepared by weighing 2.4 mg of DPPH and transferring it to a 100 mL flask, filling the volume with ethanol, and covering the flask with aluminum foil. This solution was stirred in a mixer for 30 min so that 0.06 mM DPPH stock solution was achieved. Next, in a 100 mL volumetric flask, 10 mL of the DPPH stock solution was diluted to 90 mL with ethanol PA. After that, 0.5 mL of GPE was added to 4.5 mL of a DPPH solution in a test tube, and the reaction was allowed to proceed for 30 min. A blank solution was prepared by using ethanol P.A. only, while the control solution was prepared by using 0.5 mL of ethanol P.A. and 4.5 mL of DPPH stock solution. Absorbance at 517 nm was measured on a spectrophotometer (Femto 600 Plus, Brazil). Finally, the percentage of DPPH radical inhibition by the GPE was obtained as a response.

The GPE soluble solids content (°Brix) was measured with a refractometer (HI 96801 Refractometer).

### 2.7 Preparation of Babassu Mesocarp Starch Film Added with Optimal Grape Pomace Extract

The BS-GPE film was produced by the casting method. To this end, 7.5 g of BS film was added to 92.5 g of deionized water and 2.49 g of optimal GPE (volume of 50 mL or total mass of 33%) to give 150 g of film-forming solution. The suspension was homogenized on an Ultra-Turrax (TE-102-Turratec, Tecnal) at 8,000 rpm for 5 min, which was followed by heating to 85 °C and magnetic stirring (Fisatom, model 752, Brazil) for 1.5 h. Then, 25 g of glycerol/100 g of BS film was added to the film-forming solution, which was poured into Teflon plates and dried in an oven (Quimis, Q314M292, Brazil) at 35 °C for 12 h. The BS-GPE film was conditioned at 58% RH (saturated NaBr solution) and 25 ± 1 °C for 48 h and characterized. The BS film, without GPE, was used as control.

To produce BS-GPE straws, the BS-GPE film was rolled up by using a glass rod and a cassava starch solution, as glue, and dried in a Quimis (Q314M) forced circulation oven at 50 °C for 1 h.

### 2.8 Characterization of Films

#### 2.8.1 Mechanical Properties of the BS-GPE Film

Film thickness was measured by using an electronic digital micrometer (ZAAS Precision) at six positions. Mechanical tests were carried out by employing a TA TX Plus texturometer (TA Instrument, UK). The BS-GPE film tensile strength (TS), elongation at break (EB), and Young’s modulus (YM) were measured according to the ASTM D882-18 method.^46^ The values represent the average of five determinations. The film dimensions were 2.54 cm × 10 cm. The film samples were fixed to the texture analyzer at an initial distance of 80 mm and a testing speed of 1 mm/s. TS and EB were directly obtained from the stress vs strain curve. YM was calculated by determining the slope of the initial linear portion of this curve with the help of the V.1.22 Texturometer software (SMS).

#### 2.8.2 Functional and Active Properties of the BS-GPE Film

The water solubility and moisture content of the BS-GPE film were measured by gravimetric methods. Water solubility was calculated as the percentage of dry matter in the BS-GPE film that had been solubilized after the film was immersed in water at 25 ± 2 °C for 24 h, as described by Tapia-Blácido et al.^47^ The moisture content was determined by drying the BS-GPE film in an oven at 105 °C until the film reached a constant mass (∼24 h) according to ASTM method D644-99.^48^ The water contact angle (WCA) of the BS-GPE film was determined on an OCA 20 instrument (Dataphysics, Germany) equipped with the GIMP 2.6.8 software. WCA is the angle that is formed between the water drop and the film surface.^49^ To measure WCA, 10 μL of distilled water was deposited on the surface of the BS-GPE film. The measurements were carried out six times.

The TPC and AOA of the BS-GPE film were determined from aqueous extracts of the BS-GPE film by using the methods described in Section 2.6.

#### 2.8.3 X-ray Diffraction (XRD) of Films

X-ray diffraction analysis was carried out using a Bruker AXS D2 Phaser diffractometer (Bruker, USA, Model D2 Phaser). A coupled theta/2theta scanner was used, with a scanning range of 5° to 50° at intervals of 0.02° in 0.2 s. The scanning speed was 5.6°/min with a sample rotation of 15.0 rpm. No prior sample preparation. Pieces of 2 cm were cut from the films for analysis.

#### 2.8.4 Thermogravimetry (TGA) of Films

The films were analyzed using thermogravimetry on a TGA-Q500 thermogravimetric analyzer (TA Instruments, USA, Model Q600 Simultaneous TGA/DTG/DSC). The film was analyzed under a nitrogen atmosphere at temperatures ranging from 10 to 700 °C with a heating rate of 10 °C/min. The thermal stability of the samples was evaluated using thermogravimetric (TG) and TG derivative (DTG) curves.

#### 2.8.5 Fourier Transform Vibrational Spectroscopy (FTIR) of Films

The analysis of vibrational spectroscopy in the infrared region (FTIR by Fourier transform) was carried out by reflectance using an ATR kit on Shimadzu’s IRPrestige-21 equipment (Shimadzu, Japan, model IRPrestige-21). As solid materials were used, there was no need for prior sample preparation.

#### 2.8.6 Scanning Electron Microscopy (SEM) of Films

Film samples were prepared by fixing them to aluminum sample holders with carbon-based conductive double-sided tape and then coating them in gold using a Bal-Tec mod. An SCD 050 sputter coater (Liechtenstein) was used for two periods of 200 s each. The microstructure of the films was analyzed using a Shimadzu model SS-550 (Kyoto, Japan) at 20 kV or 10 kV.

### 2.9 Evaluation of the Smart Property of the BS-GPE Film—pH Indicator

The BS-GPE film was exposed to solutions with pH of 1, 5, 7, 9, or 13. Color changes were observed after 1 min. Colorimetric characterization of the film was carried out by using the Trigit software.^50^ The film was photographed with a Samsung Galaxy A53 smartphone. Three colorimetric measurements were taken in three different areas of the BS-GPE film. To calculate Δ*E*, the standard sample of white bond paper (a* = 3.840, b* = 7.630, L* = 69.780) was used as the basis for the calculation, under the same photographic conditions (lighting and camera) as those used for the samples. The calculation was performed according to eq 3:

3 $$\Delta E=\sqrt{\Delta L^{2}+\Delta a^{2}+\Delta b^{2}}$$

### 2.10 BS-GPE Application as Eco-Smart Straw

The BS-GPE film was tested as an eco-straw in sugar-free cola soda. Specifically, the integrity of the BS-GPE film was evaluated after it was immersed in sugar-free cola soda for 0, 5, 10, 15, 20, 25, 30, 60, or 120 min. The liquid was analyzed for soluble solids (°Brix), which were directly measured on an HI 96801 refractometer.

### 2.11 Statistical Analysis

The data were statistically analyzed by using one-way ANOVA. A Tukey’s test was performed to evaluate how the GPE solvent and granulometry affected the GPE bioactive properties. Differences between the BS and GPE-BS films were also analyzed. Statistical analyses of the data were performed by one-way ANOVA at a 5% significance level (*p* < 0.05); the Statistica 14.0 software (StatSoft, USA) was employed.

## 3 Results and Discussion

### 3.1 Chemical Characterization of Babassu Mesocarp Starch and Grape Pomace

The babassu mesocarp starch (BS) extraction yield was 52 ± 2%. BS contained 9.93 ± 0.07% moisture, 1.51 ± 0.01% ash, 0.38 ± 0.0% lipids, 1.55 ± 0.01% proteins, and 96.16 ± 0.12% carbohydrates. The amylose content was 367.19 ± 2.83 mg/g of starch. BS had a TPC of 93.67 ± 0.01 mg of GAE/L and an AOA of 79.94 ± 0.01%, as measured by DPPH. BS had similar moisture, ash, and protein contents to the contents found by Maniglia and Tapia-Blácido^35^ for this material. Babassu mesocarp could also contain some chemical elements, including iron, copper, and zinc.^51^ The ionic forms of these elements could help BS to capture free radicals. In addition to these elements, babassu mesocarp might contain potassium, calcium, magnesium, phosphorus, mercury, and iodine.^52^ Valorizing byproducts with considerable antioxidant activity and food potential is in line with green chemistry principles, especially principle 7, which concerns the use of renewable raw materials.^53^

Figure 1 shows BS micrographs at 1000× magnification. The starch granules were oval; the average granule size was approximately 10 μm. Marta et al.^54^ observed that banana starch granules have a size of this same magnitude, but they present a more elongated oval morphology. In turn, Yilmaz and Tugrul^55^ found that corn starch granules morphologically resemble BS.

**Figure 1:**
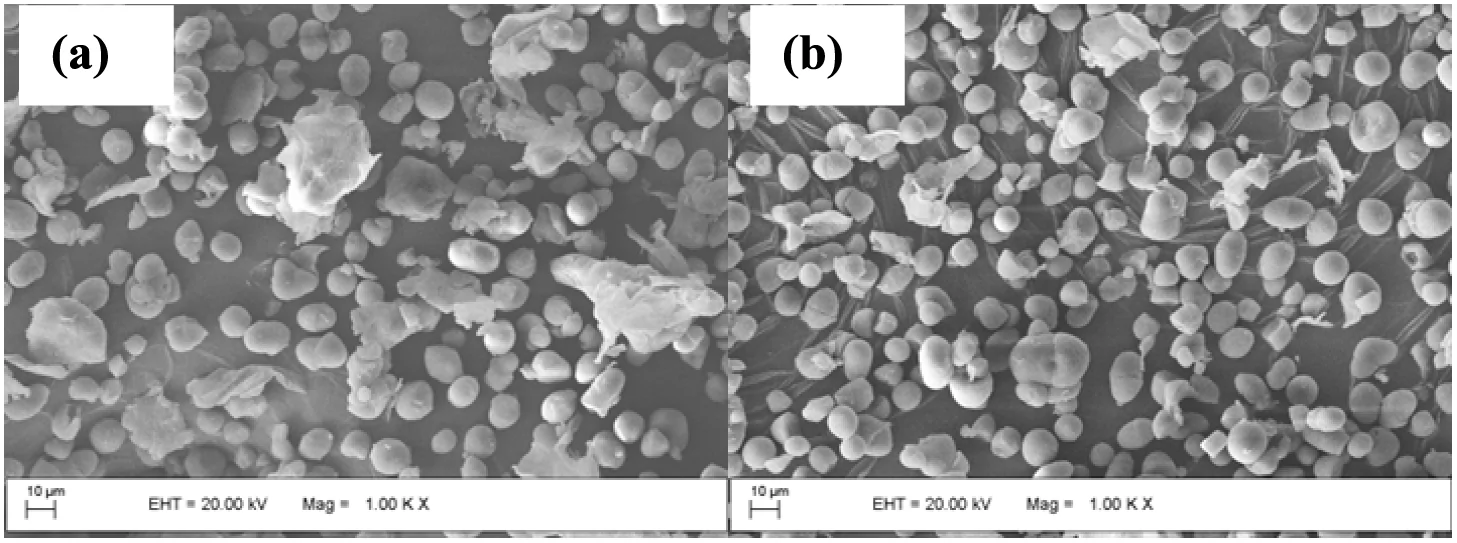
SEM micrograph images of (a) babassu mesocarp flour and (b) babassu mesocarp starch.

Grape pomace (GP) contained 13.94 ± 0.13% moisture, 4.36 ± 0.07% ash, 2.0 ± 0.21% lipids, 5.03 ± 0.03% proteins, and 87.75 ± 0.52% carbohydrates. GP had higher moisture content and lower ash content than grape pomaces reported by other authors.^56,57^ Compared to these studies, GP also had different lipid and protein contents, which could be due to differences in cultivars, climatic conditions, soil, and other technological factors associated with the unitary wine production processes. GP contained mostly carbohydrates, such as pectin and fibers.

After its centesimal characterization, GP was divided into three distinct fractions: G fraction (*in natura*, without grinding), M fraction (>45 Tyler-Mesh), and P fraction (<45 Tyler-Mesh). The M was the fraction that was retained, and P was the fraction that passed through the 45-mesh sieve. All the GP fractions had an intense color, which indicated that they contain bioactive compounds. The particle size distribution of the GP fractions as well as their appearance can be seen in Supporting Information Figure S1. The size of the particles of material is important for extracting bioactive compounds. P had a polymodal distribution, and the three peaks corresponded to average particle sizes of 8.14, 36.23, and 69.62 μm. M also presented polymodal distribution, and the nine peaks corresponded to average particle sizes of 194.23, 282.07, 449.67, 594.87, 786.95, 863.88, 1041.05, 1254.55, and 1377.2 μm. Thus, M contained particles larger than 1000 μm, while P presented smaller particles. It was not possible to analyze the particle size distribution of the unground GP fraction (G) due to technical limitations of the equipment. Brito et al.^58^ pointed out that fractions with particle sizes smaller than 150 μm contain more proteins and lipids, while fractions with particle sizes larger than 425 μm contain more fibers, cellulosic compounds, and lignins.

### 3.2 Effect of Solvent Type and Grape Pomace Granulometry on the Extraction of Bioactive Compounds from Grape Pomace

As shown in Table 1, acidified solvents, particularly Et_Ac_, produced GPE with a high TAC (271.52 mg of Cya-3-Glu equivalent/L), followed by H_2_O_Ac_ (79.82 mg of Cya-3-Glu equivalent/L). Despite their high TAC value, these extracts exhibited low antioxidant activity (11.16% and 33.68%). This behavior differed from what was expected. This may be due to the acidic nature of the extract. Anthocyanins become highly stable in flavin ion form. This form has a positive charge on the oxygen atom in the central ring of the carbon chain, promoting aromaticity.^83^ Consequently, the protonated form is less reactive with the DPPH radical, which decreases its antioxidant activity in an acidic medium. When either ethanol (EtOH) or water (H_2_O) was used as the extraction solvent, the resulting GPE had a similar total acid content (TAC) of approximately 80 mg/L of cyanidin-3-glucoside equivalent and a high AOA (74.40–78.59%). However, extraction with H_2_O provided a GPE with a higher TPC (151.68 mg of GAE/L) than extraction in an acidic medium (EtAc or H_2_OAc).^84^

**Table 1:** Bioactive Compound Content, Antioxidant Activity, and Soluble Solids Content in Grape Pomace Extracts Obtained with Different Solvents^a^

Extraction solvent	TAC (mg of cya-3-glu equivalent/L)	TPC (mg of GAE/L)	AOA (%)	Soluble solids (°Brix)
H_2_O	79.82 ± 3.54^c^	151.68 ± 0.41^a^	74.40 ± 0.14^a^	6.2 ± 0.2^c^
Et	88.17 ± 0.47^c^	140.52 ± 3.69^b^	78.59 ± 0.14^a^	21.4 ± 0.5^b^
H_2_O_Ac_	161.65 ± 3.78^b^	135.30 ± 12.00^c^	33.68 ± 7.84^b^	6.8 ± 0.1^c^
Et_Ac_	271.52 ± 3.78^a^	136.46 ± 4.51^c^	11.16 ± 1.41^c^	24.7 ± 0.3^a^

a Different lowercase letters in the same column indicate a significant difference between the grape pomace extracts obtained with different solvents according to Tukey’s analysis (*p* < 0.05). **TAC**, total anthocyanin content; **TPC**, total phenolic compounds; **AOA**, antioxidant activity. Ac = acidified.

In addition, the GPE obtained with H_2_O had a lower soluble solids content than that obtained with Et. This difference can be attributed to their respective dielectric constants (80.1 for H_2_O and 24.5 for Et). This result indicates a disadvantage of the ethanol medium compared to the aqueous medium, since ethanol reduces the solubility of bioactive compounds, suggesting that water offers greater solvation capacity for the compounds present in the GPE. Pintać et al.^59^ evaluated different solvents to extract bioactive compounds from grape pomace and observed that 80% methanol extracts polyphenols in a purer form more effectively. In turn, Natolino et al.^60^ tested water, ethanol, and a 1:1 (v/v) water/ethanol mixture as solvents for classic extraction and subcritical solvent extraction of grape pomace and achieved better results with the solvent mixture. Other extraction methods have also been tested to extract bioactive compounds from grape pomace or fruit, including pressurized liquid and ultrasound with different classes of solvents or at different temperatures.^61–63^ Given that GPE could be used as an additive in the food industry and bearing the green chemistry principles in mind (particularly principles 3 (use less dangerous processes), 4 (design safe chemistry), 5 (use safe solvents), and 12 (prevent accidents)),^53^ we chose to employ H_2_O as the extraction solvent, while H_2_OAc was not chosen as the solvent to preserve the “smart” properties of the anthocyanins. In an acidic environment, the anthocyanins would take longer to change color due to the acid–base equilibrium shift caused by the added acid. Furthermore, as previously mentioned, this acid-based extraction method is not considered to be environmentally friendly.

Table 2 lists the composition of grape pomace extract produced from GP fractions M, P, and G (nonmilled GP). The extract obtained from GP fraction M had high bioactive compound content (103.54 mg of GAE/L and 80.41 mg of cya-3-glu equivalent/L) and the highest AOA. Meanwhile, GP fraction G yielded an extract with lower TPC and AOA than the GP fraction M. This suggests that a light grind is required to release the bioactive compounds from the GP. In this sense, the GP containing particles measuring ∼1,000 μm in size (fraction M) was better suited to extracting bioactive compounds. Conversely, the GP extract produced from small particles (fraction P) had the lowest TAC (70.89 mg cyanidin-3-glucoside equivalent/L) and AOA (66.95%), with a similar TPC (100.95 mg GAE/L). Therefore, GP with a small particle size should be avoided, since anthocyanins may be retained in GP fractions containing larger particles.

**Table 2:** Characterization of Extracts Obtained from Grape Pomace with Different Granulometry^a^

Grape pomace fraction	TAC (mg of cya-3-glu equivalent/L)	TPC (mg of GAE/L)	AOA (%)	Dry matter (%)	Soluble solids (°Brix)
P	70.89 ± 0.35^b^	100.95 ± 0.00^b^	66.95 ± 0.00^c^	5.06 ± 0.01^a^	5.9 ± 0.0^b^
M	80.41 ± 0.35^a^	103.54 ± 0.27^a^	91.52 ± 0.00^a^	4.98 ± 0.30^a^	7.1 ± 0.0^a^
G	81.32 ± 0.24^a^	86.14 ± 0.18^c^	79.88 ± 0.00^b^	2.92 ± 0.03^b^	4.1 ± 0.0^c^

a P: Fraction < 45 mesh, M: Fraction > 45 mesh, G*: In natura* grape pomace. TAC: total anthocyanin content, TPC: Total phenolic compounds, and AOA: Antioxidant activity. Different lowercase letters in the same column indicate a significant difference between the grape pomace fractions according to Tukey’s analysis (*p* < 0.05).

### 3.3 Optimizing Extraction of Bioactive Compounds from Grape Pomace

To optimize the extraction of bioactive compounds from GP, we used GP fraction M and H_2_O as solvent based on the results obtained in the previous section. Table 3 summarizes TAC, TPC, and AOA of GPE obtained according to CCRD 2^2^.

**Table 3:** Central Composite Rotatable Design 2^2^ Used to Optimize the Extraction of Bioactive Compounds from Grape Pomace^a^

Test	X_1_ (Temperature, °C)	X_2_ (Time, h)	TAC (mg of cya-3-glu equivalent/L)	TPC (mg of GAE/L)	Antioxidant activity (%)
1	–1 (45)	–1 (1.5)	93.62 ± 0.83	120.46 ± 0.72	75.90 ± 0.00
2	1(65)	–1 (1.5)	116.81 ± 1.54	140.26 ± 0.10	70.82 ± 0.14
3	–1 (45)	1 (2.5)	80.86 ± 2.13	136.70 ± 0.20	75.30 ± 1.41
4	1 (65)	1 (2.5)	128.41 ± 1.89	142.06 ± 0.31	68.43 ± 0.70
5	–1.41 (41)	0 (2)	78.32 ± 1.89	127.57 ± 0.41	76.39 ± 0.58
6	1.41 (69)	0 (2)	137.35 ± 0.59	151.04 ± 1.74	63.66 ± 1.31
7	0 (55)	–1.41(0.8)	87.59 ± 1.30	129.59 ± 0.72	74.02 ± 0.73
8	0 (55)	1.41 (3.2)	87.84 ± 3.42	132.49 ± 0.10	73.61 ± 0.44
9	0 (55)	0 (2)	100.53 ± 0.71	137.57 ± 0.41	71.18 ± 0.15
10	0 (55)	0 (2)	99.53 ± 4.49	136.12 ± 0.20	72.21 ± 1.02
11	0 (55)	0 (2)	102.53 ± 1.65	136.84 ± 0.10	72.31 ± 0.29

a **TAC**, total anthocyanin content; **TPC**, total phenolic compounds.

Table 3 shows that changing the extraction temperature (from 41 to 69 °C) and time (from 0.8 to 3.2 h) resulted in GPE with TAC ranging from 80.86 to 137.35 mg of cya-3-glu equivalent/L, TPC ranging from 120.46 to 151.04 mg of GAE/L, and AOA ranging from 63.66 to 76.39%. Specifically, extraction performed at 69 °C for 2 h (test 6) gave GPE with the highest TAC (137.3 ± 0.6 mg of cya-3-glu equivalent/L) and TPC (151.0 ± 1.7 mg of GAE/L), whereas extraction performed at 41 °C for 2 h (test 5) provided GPE with low TAC (78.3 ± 1.9 mg of cya-3-glu equivalent/L) and TPC (120.5 ± 0.7 mg of GAE/L). However, in the latter case, GPE exhibited higher AOA (75.9%) than GPE obtained in test 6.

At high extraction temperatures, anthocyanins and phenolic compounds polymerize to form proanthocyanidins and/or other polymeric compounds, such as anthocyanin-phenolic and tannin copigments. These compounds prevent the actual AOA of the extract from being determined by the DPPH method.^64–66^ The DPPH assay detects them in their polymeric form, the steric hindrance of which reduces the radical scavenging. Furthermore, using ethanol as the solvent in the DPPH assay hinders the complete solubilization of the GPE compounds. This is because the dielectric constant of ethanol is 24.5, compared with 80.1 for water. Meanwhile, the anthocyanin quantification assay is performed in an acidic medium, which breaks down these polymeric tannins into anthocyanin monomers, enabling the total anthocyanin content to be determined.^75–77^

Figure 2 depicts the Pareto charts obtained for the GPE TAC, TPC, and AOA and shows how the independent variables (X_1_, extraction temperature; X_2_, extraction time) affected the responses. The further to the right, the more pronounced the effect of each independent variable (linear, quadratic, and interaction parameters).^67^

**Figure 2:**
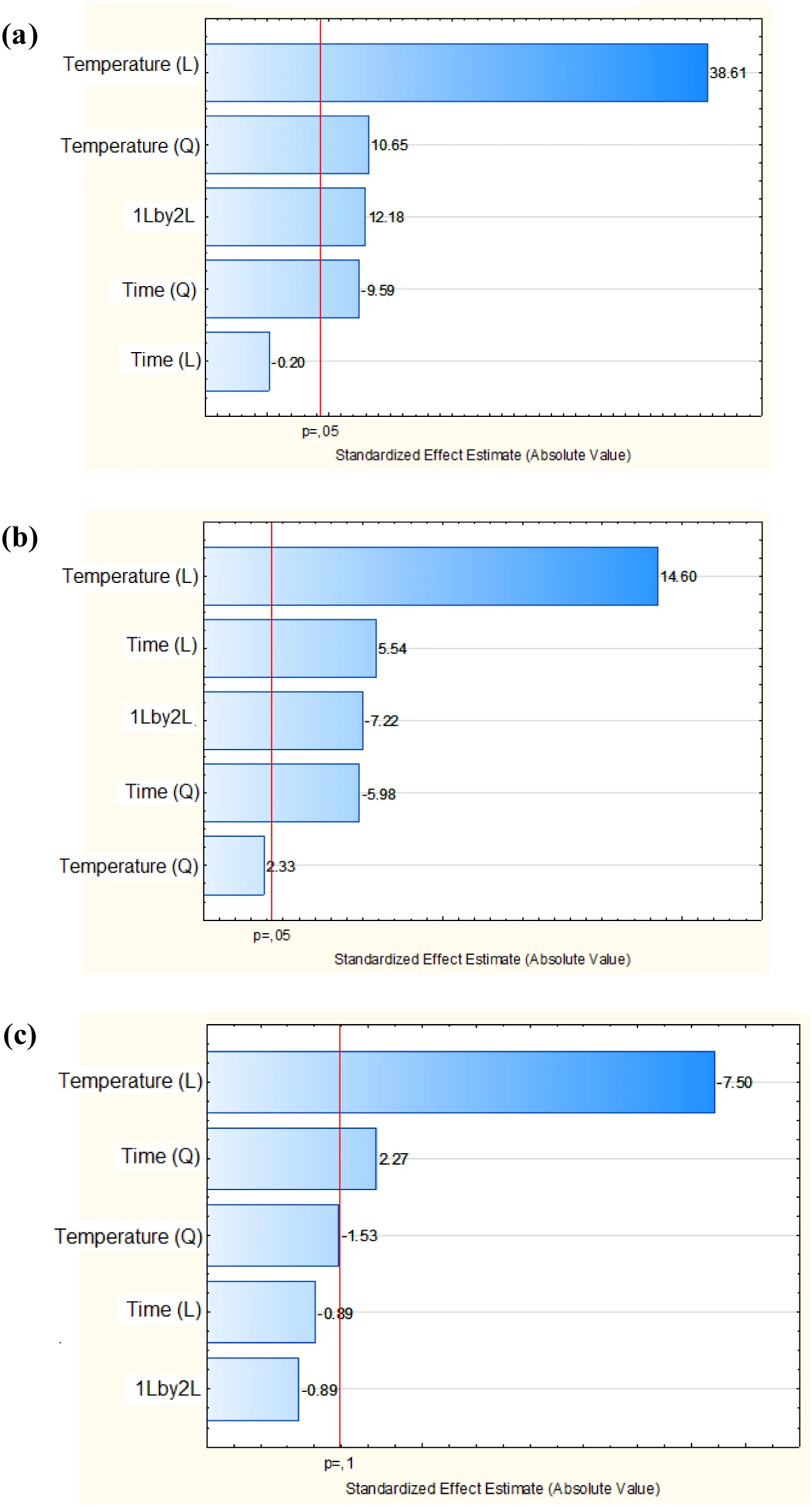
Pareto charts for the dependent variables (responses): GPE (a) total anthocyanins content (TAC, in mg of cyanindin-3-glucose equivalent/L), (b) total phenolic compound content (TPC, in mg of gallic acid equivalent/L), and (c) antioxidant activity (AOA, in %).

Figure 2a shows that the linear and quadratic terms of the extraction temperature (X_1_ and X_1_^2^) positively affected the GPE TAC. Increasing the extraction temperature increased TAC by 38.6 mg of cya-3-glu equivalent/L. The quadratic term of extraction time (X_2_^2^) negatively affected TAC, while the X_1_X_2_ interaction positively impacted the TAC. Zhou and Yang^65^ observed that increasing extraction temperature increases TAC, but longer extraction time (>50 min) causes anthocyanins to be oxidized, hydrolyzed, or polymerized.

Figure 2b shows that the linear term of the extraction temperature (X_1_) also positively affected the GPE TPC. However, extraction time affected TPC to a larger extent than TAC given that its linear and quadratic terms (X_2_ and X_2_^2^) were significant for TPC. The linear term of extraction time (X_2_) positively affected TPC, so longer extraction time (−1 to +1) allowed more phenolic compounds to be extracted from GP. Nevertheless, the quadratic term of the extraction time (X_2_^2^) negatively affected TPC. In addition, in contrast to TAC, the X_1_X_2_ interaction negatively affected the TPC.

Figure 2c shows that the linear term of the extraction temperature (X_1_) negatively affected the GPE AOA. Thus, increasing the extraction temperature (from −1 to +1) decreased AOA because high temperature causes anthocyanins to be oxidized or polymerized so that the conjugated double bonds are cleaved and the resonance effect is lost. This prevents free radicals from being stabilized, which translates into lower AOA.^64,66^ On the other hand, extraction time (X_2_) did not affect AOA, so extraction time ranging from 0.8 to 3.2 h did not impair the GPE antioxidant properties.

Table 4 displays the ANOVA (analysis of variance) and regression coefficients for the GPEs’ TAC, TPC, and AOA. The fitted second-order mathematical models for these responses were significant and predictive, as attested by the F-test (F_c_ ≫ F_tab_, and F_lack of fit_ < F_tab_).

**Table 4:** ANOVA and Regression Coefficients of CCRD 2^2^ Conducted for Grape Pomace Extraction

Coefficient	TAC (mg of cya-3-glu equivalent/L)	TPC (mg of GAE/L)	AOA (%)
Βo	100.83	137.93	71.90
Linear			
β_1_	19.30	7.30	–3.75
β_2_	–0.10^a^	2.77	–0.44^b^
Quadratic			
β_1_^2^	5.32	1.16^a^	–0.77
β_2_^2^	–4.80	–3.33	1.14
Interaction			
β_1_β_2_	6.09	–3.61	–0.45^b^
F_calculated_	41.24	22.08	31.19
F_tabulated_	4.53	4.53	4.35
F_lack of fit_	13.19^a^	19.13^a^	4.50^b^
F_tabulated_	19.12	19.30	9.29
R^2^	0.97	0.94	0.93

a Indicates nonsignificance at 95% confidence intervals.

b Indicates nonsignificance at 90% confidence intervals.

The models had high R^2^ (from 0.93 to 0.97), which indicated a good fit with reasonable agreement. The regression mathematical models are shown in eqs 4, 5, and 6:

4 $$\mathrm{Anthocyanins (mg of cya‐3‐glu equivalent/L)}=100.8328+19.3041X_{1}+5.327{X_{1}}^{2}-4.7951{X_{2}}^{2}+6.0914X_{1}X_{2}$$

5 $$\mathrm{TPC (mg of GAE/L)}=137.9331+7.3039X_{1}+2.7727X_{2}-3.3304{X_{2}}^{2}-3.6087X_{1}X_{2}$$

6 $$\mathrm{AOA (\%)}=71.89809-3.74984X_{1}-0.76881{X_{1}}^{2}+1.13970{X_{2}}^{2}$$

### 3.4 Response Surfaces and Optimal Conditions for Extracting Active Compounds from Grape Pomace

Figure 3 shows the response surfaces generated from the second-order mathematical models obtained for the GPE TAC, TPC, and AOA (eqs 3, 4, and 5, respectively). The TAC response surface shows that extraction temperature (X_1_) had the greatest effect on this response (Figure 3a). The high TAC region corresponded to high extraction temperature (>65 °C) and long extraction time >1.5 h. This behavior was different for TPC (Figure 3b). GPE with high TPC could be obtained at temperatures higher than 60 °C within a large range of extraction time. However, low extraction temperatures (<45 °C) provided GPE with better AOA (Figure 3c), which was consistent with the extraction temperature negatively affecting TAC, which could lead anthocyanins to be degraded or polymerized.

**Figure 3:**
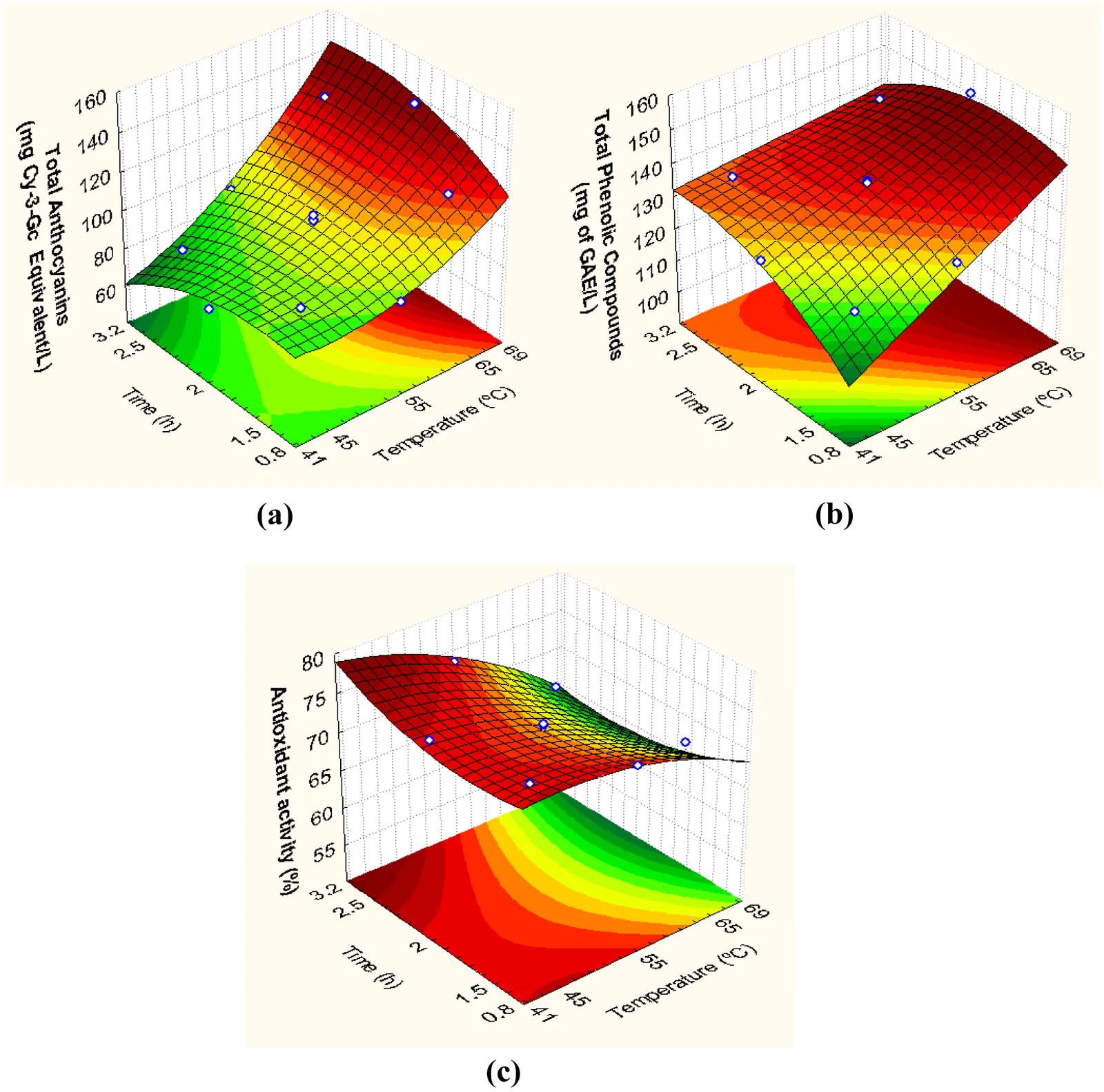
Response surfaces for grape pomace extract: (a) total anthocyanin content, (b) total phenolic compound content, and (c) antioxidant activity.

We used second-order mathematical models (eqs 4 and 5) to determine the optimal temperature and time for achieving high TAC and TPC values in GPE, using the desirability function. The AOA response (eq 6) was not included in the desirability function, as this value does not agree with the bioactive compound content. The optimal extraction conditions were 69 °C (X_1_ = 1.41) for 2 h (X_2_ = 0). We applied these conditions to extract the bioactive compounds from the GP. Table 5 shows the predicted values of TAC, TPC and AOA, as calculated by the mathematical models presented in eqs 4–6. A comparison of the predicted and experimental values of TAC, TPC, and AOA showed a low relative deviation of −1.60%, 9.61%, and 5.96%, respectively, indicating good agreement between the mathematical models and the experimental data.

**Table 5:** Comparison between the Predictive and Experimental Results for the Optimized Grape Pomace Extract^a^

Value	TAC (mg of cya-3-glu equivalent/L)	TPC (mg of GAE/L)	AOA (%)
Predictive	138.64	148.23	65.08
Experimental	136.46	163.99	69.21
Relative deviation (%)	–1.60	9.61	5.96

a Relative deviation (%) = [(experimental value – predicted value)/experimental value] × 100. TAC: Total anthocyanins content, TPC: Total phenolic compound content; AOA: Antioxidant activity.

Other authors found that the optimal conditions to extract anthocyanins (193.54 mg of cya-3-glu equivalent/100 g of grape pomace) were an extraction temperature of 63.8 °C for 48 h when using the ultrasound-assisted method and ethanol as the solvent.^65^ Our result was close to that, with the advantage that we did not use an ultrasound-assisted methodology and that we employed water as the extraction solvent, which fits into the sustainability and green chemistry concept.

### 3.5 SEM of BS and BS-GPE Films

Figure 4 shows the micrographs of BS and BS-GPE films, revealing the presence of microcrystalline particles on the surface of the BS-GPE film (marked in circles), possibly a tartrate crystal. The crystals formed during the drying process of the film and were not incorporated into the polymeric matrix. Moreover, the surface of both films was rough and featured oval patterns that may represent gelatinized babassu starch granules. Figure 4B.1 illustrates the presence of gelatinized starch granule agglomerates on the surface of the BS-GPE film.

**Figure 4:**
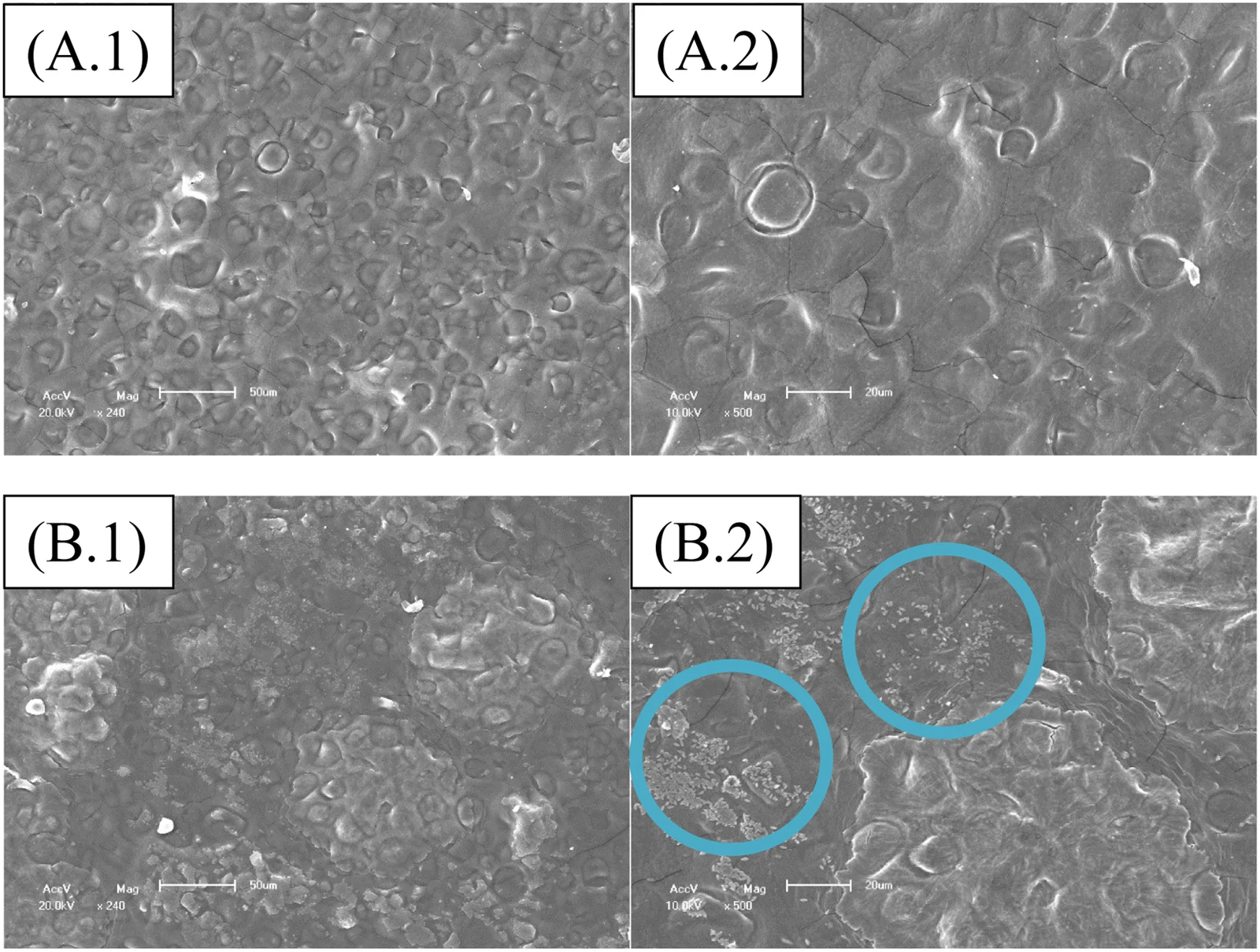
Micrographs of the samples: (A.1) BS film at 240× magnification, (A.2) BS at 500× magnification, (B.1) BS-GPE film at 240× magnification, and (B.2) BS-GPE at 500× magnification.

### 3.6 Optical, Mechanical, and Functional Properties of Films

GPE is a water-based extract containing high levels of bioactive compounds, particularly anthocyanins, which can be used in the food industry. Its solubility in starch suspensions enables the production of smart and active films. Figure 5 shows the visual appearance of the BS and BS-GPE films. The a* color parameter increased after GPE was added to the BS film, which indicated a more reddish color characteristic of GPE. The L* parameter increased slightly after the GPE was added to the BS film. However, both the BS and BS-GPE films presented low luminosity and were colorful.

**Figure 5:**
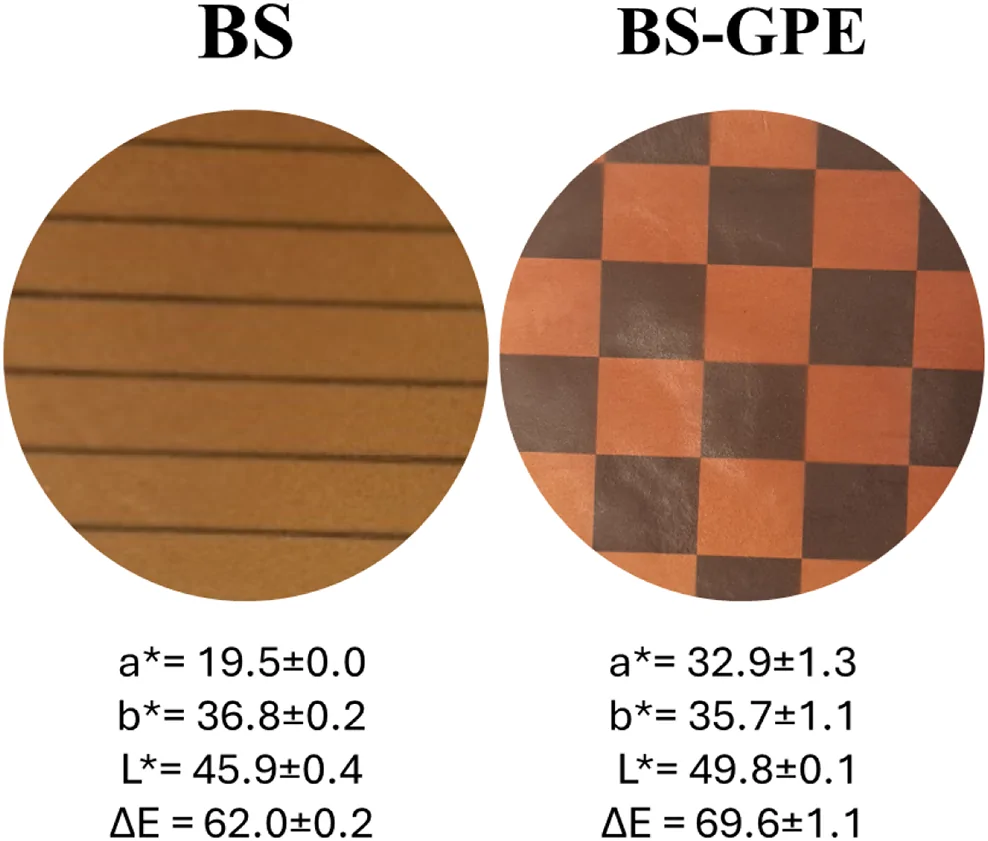
Visual aspect of babassu mesocarp starch (BS) film without and with added grape pomace extract (GPE).

Table 6 shows the BS and BS-GPE film properties. The BS film had higher TS (8.32 ± 0.65 MPa) and YM (191.93 ± 19.70 MPa) than the BS-GPE film. Meanwhile, GPE addition increased the EB of the film. Anthocyanins and phenolic compounds can act as plasticizers by accommodating between the BS polymer chains, thereby decreasing the mechanical resistance and increasing the EB of the BS-GPE film.

**Table 6:** Properties of Babassu Mesocarp Starch Film with and without Added Grape Pomace Extract

Property	Babassu starch film	Babassu starch film added with grape pomace extract
Thickness (mm)	0.09 ± 0.01^b^	0.14 ± 0.02^a^
Tensile strength (MPa)	8.32 ± 0.65^a^	3.54 ± 0.07^b^
Elongation at break (%)	32.09 ± 3.49^c^	36.53 ±1.30^a^
Young’s modulus (MPa)	191.93 ± 19.70^a^	31.27 ± 0.68^b^
Moisture (%)	23.52 ± 0.19^b^	28.20 ± 1.86^a^
Solubility (%)	40.79 ± 0.96^b^	46.57 ± 0.37^a^
Contact angle (°)	28.81 ± 0.93^b^	58.35 ± 3.76^a^
DPPH (%)	67.94 ± 1.08^b^	71.85 ± 0.23^a^

Regarding functional properties, the BS-GPE film had higher moisture content (28.20 ± 1.86%) and solubility (46.57 ± 0.37%) than the BS film. This indicates the presence of free hydroxyl groups that can interact with water molecules, thereby increasing the moisture content and solubility. However, the BS-GPE film had a lower surface hydrophilicity than the BS film, as the contact angle increased from 28.81° to 58.35°. This can be justified by the micrograph of the surface shown in Figure 4, since the BS-GPE film has tartrate crystals and a rougher surface with agglomerates, which decrease the wettability of the film.

GPE addition increased the BS film’s AOA. From the point of view of green chemistry principles, the BS-GPE film complied with the principles of less-hazardous synthesis (principle 3) because it was obtained by the casting method, which is safer (principle 4). In addition, the BS-GPE film was prepared from renewable raw materials (principle 7), which allowed agro-industrial waste to be reused and valorized. Finally, the BS-GPE film can potentially be applied in the food industry.^53^

### 3.7 XRD of BS and BS-GPE Films

The results of the comparison between the BS control films and the BS-GPE films indicate significant differences in their crystallinity. Figure 6 shows the diffractograms of these two materials. The main peaks for the control BS are near 17°, 19°, and 22°, indicating peaks characteristic of type C starch, consistent with the peaks found by Maniglia et al.^28^ Meanwhile, the peak at 24.5° distinguishes the control BS from BS-GPE, which may indicate the presence of tartrate crystals derived from grape pomace.^72^ Adding GPE to the film resulted in an ∼8% reduction in its crystallinity index, which can be explained by the plasticizing effect of phenolic compounds, such as anthocyanins. This is consistent with the results of the mechanical properties testing.

**Figure 6:**
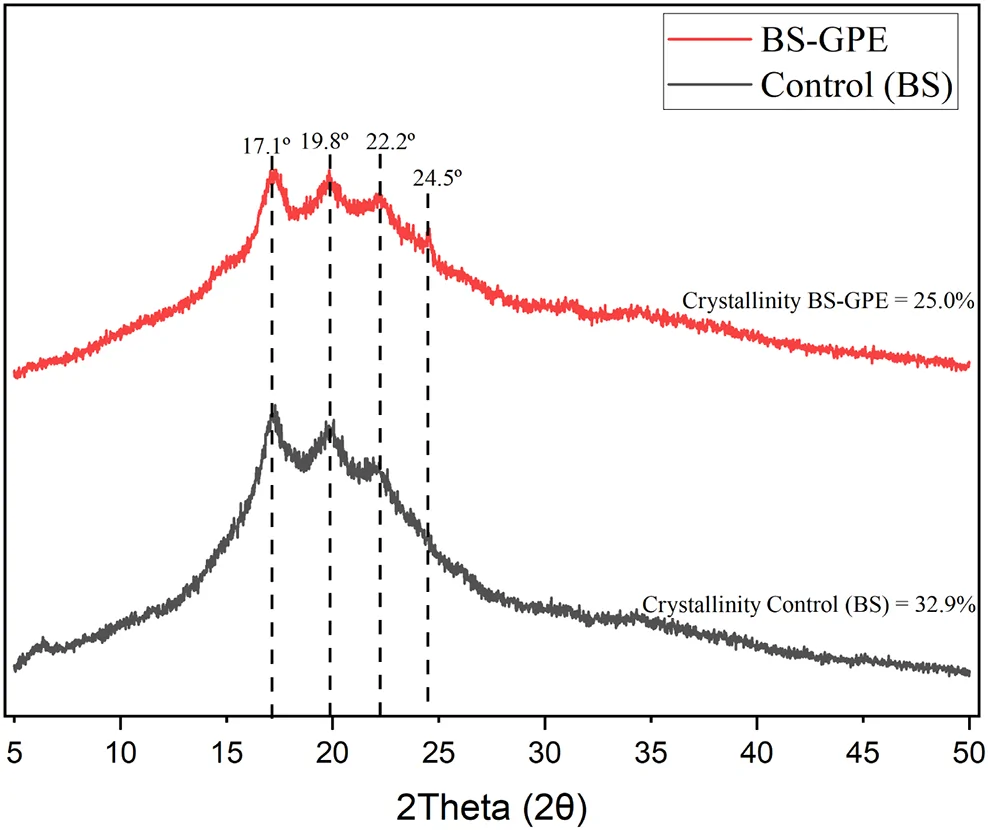
Diffractogram of the BS and BS-GPE film samples.

### 3.8 TGA of BS and BS-GPE Films

In terms of the films’ thermal properties (Figure 7), the control BS sample exhibits a first peak in mass loss near 100 °C, as does the BS-GPE film, though to a lesser extent, representing the loss of residual moisture from the material. A second peak in mass loss can be observed for BS-GPE at ∼200 °C, which may represent the decomposition of smaller bioactive compounds, while a peak of higher intensity at ∼300 °C is observed for the BS control and one of relatively lower intensity for BS-GPE, suggesting the decomposition of the material’s three-dimensional structure and its inter- and intramolecular interactions. This same two-phase decomposition pattern of grape pomace was observed by Jansen et al. (2024).^73^

**Figure 7:**
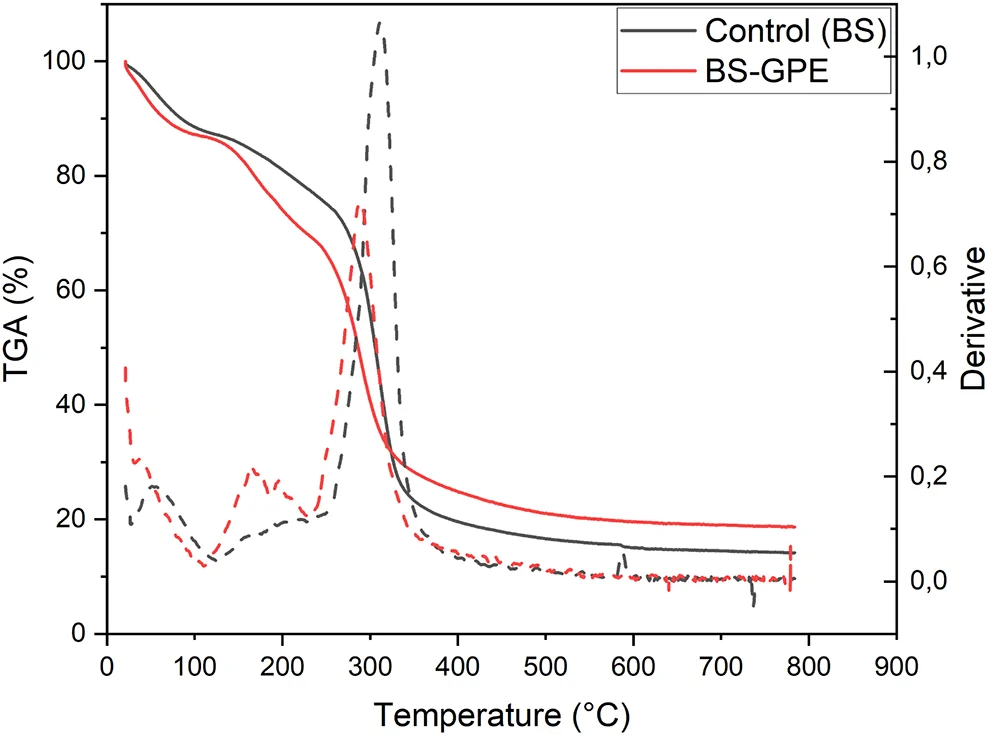
Thermogravimetric analysis of the BS and BS-GPE films and their respective derivatives.

### 3.9 FTIR of BS and BS-GPE Films

Figure 8 shows the similar spectra for BS and BS-GPE films. It was observed to peak at 3345 cm^–1^, which corresponds to O–H stretching and the hydrogen bonds formed by the amylose-amylopectin, amylopectin/amylose-anthocyanins/phenolics chains. Peak at 2935 cm^–1^ corresponds to C–H stretching, representing the d-glucose monomer that makes up the starch. At 1650 cm^–1^, the signal from the material’s amorphous region is observed due to the stretching of H–O–H bonds. The region from 1200 to 900 cm^–1^ represents the fingerprint region of starch.^74^

**Figure 8:**
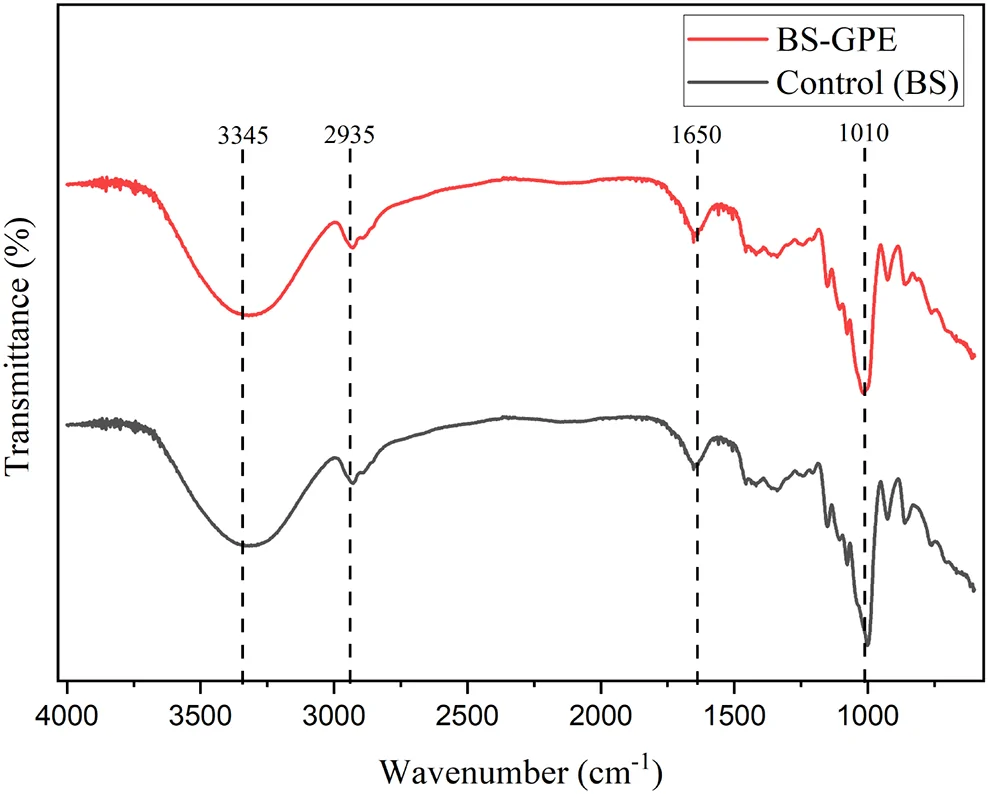
FTIR analysis of the BS and BS-GPE films.

### 3.10 Evaluation of Smart Film Property

By incorporating an extract containing anthocyanins into the BS film, we obtained the BS-GPE film, which can potentially be applied as a smart film because it can change color in response to pH variations. We investigated this property by conducting a pH sensitivity test on the GPE-BS film, as illustrated in Figure 9.

**Figure 9:**
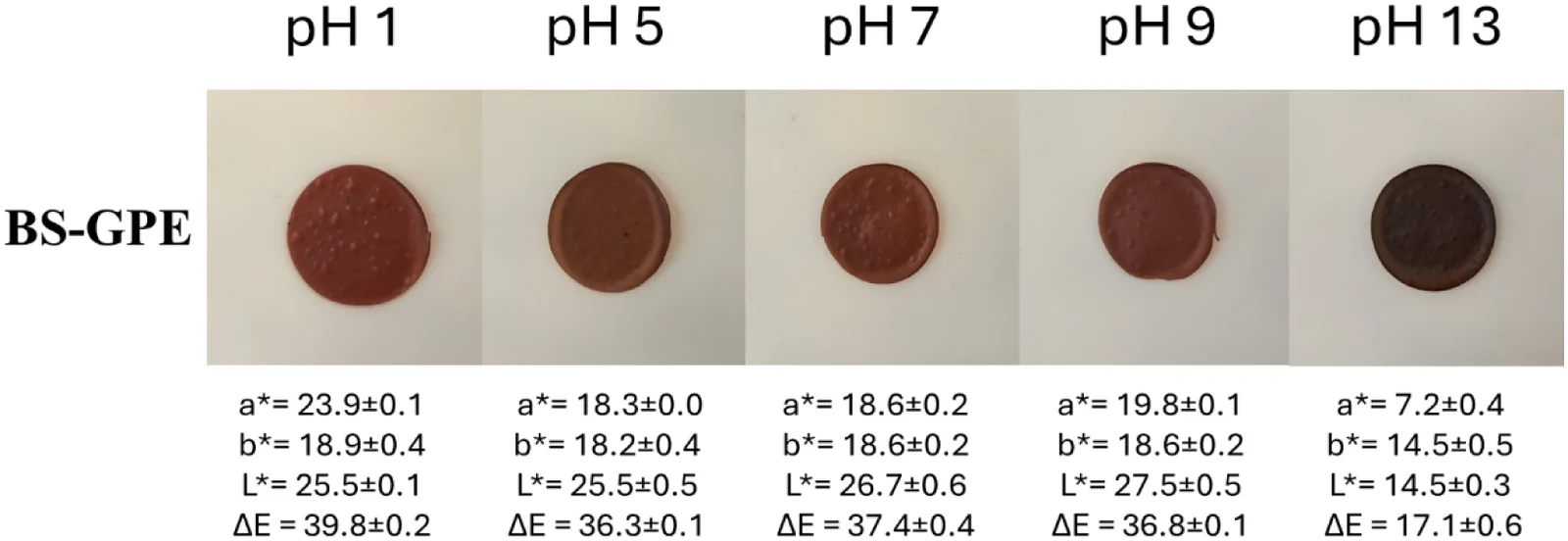
Testing a babassu starch film added with a pH-sensitive extract.

The BS-GPE film was sensitive to pH alterations: the film color changed mainly at extreme pH levels (pH = 1 and pH = 13), with the color parameter a* approaching redder tones at pH 1 and darker tones at pH 13. At pH 1, the film had a pinkish hue compared to its color at pH 7. In contrast, at pH 13, the film’s color changed to nearly black, as the anthocyanin pigmentation likely mixed with the natural color of the BS film; furthermore, the film became more brittle. This increase in brittleness was attributed to OH^–^ ions attacking the starch bonds, which weakened the film molecular structure. Similar results were reported by Vázquez et al.^68^ and Francisco et al.,^69^ who produced smart films added with grape extract with different polymeric bases. Their films also changed color, particularly at extreme pH, which coincided with the results of this study.

### 3.11 Evaluation of BS-GPE Film as Straw

Figure 10 shows that the BS-GPE film can be applied as an eco-smart straw in zero-sugar cola soft drinks: the film demonstrated excellent functionality in acidic (pH ∼ 2.5) sugar-free cola given that its structural integrity was maintained after immersion for 2 h and that no visible defects or performance issues arose during this period. Soluble solids analysis confirmed that particle release from the film was minimal (0.4 to 0.6 variation), so solubilization was negligible. These results showed that the BS-GPE straw surpassed the stability of cellulose nanofibril (CNF)-based straws and approached the performance of advanced composites like SiO_2_-reinforced CNF or self-locking chitosan.^70,71^ The combination of resistance to acid (attributed to BS matrix stability at low pH) and low solubilization suggests that the BS-GPE straw is an alternative to single-use petroleum-based plastics.

**Figure 10:**
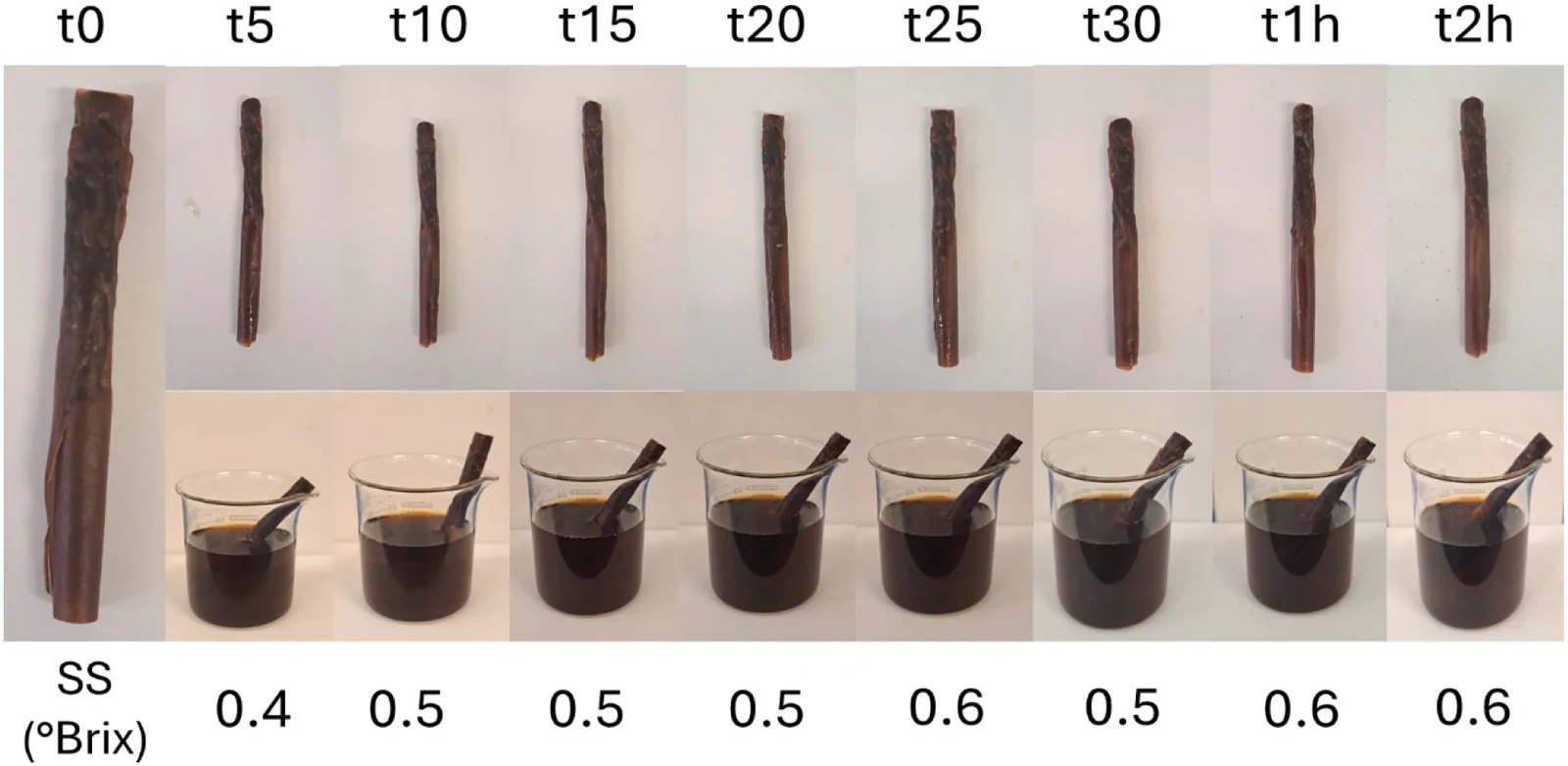
Evaluating the BS-GPE film as an eco-smart straw in sugar-free cola soft drink for 2 h.

The GPE-BS film is aligned with the principles of the circular economy because it is based on the use of agro-industrial waste.

## 4 Conclusion

We optimized the extraction of bioactive compounds from grape pomace (GP) by using water as the solvent, a 2^2^ CCRD, and response surface methodology (RSM). The optimal extraction conditions were a temperature of 69 °C and extraction for 2 h, which provided an extract (GPE) with high total anthocyanin and total phenolic compound content and acceptable antioxidant activity. The extraction temperature and time influenced the extraction of phenolic compounds and anthocyanins from GP. The predicted values obtained by using the regression mathematical models agreed with the experimental values, so the models were statistically valid. The optimal GPE can potentially be used as a smart and active agent in babassu starch (BS) film. The addition of GPE to the BS film resulted in higher elongation at break and lower mechanical strength due to the plasticizing effect of the extract. It also exhibited improved functional, active, and smart properties, as well as relatively greater hydrophobicity due to the presence of tartrate crystals and agglomerates on the film’s surface, as observed by XRD and SEM. This film can be used as an eco-friendly soda straw for at least 2 h without losing its structural integrity. This work supports several principles of green chemistry: the BS-GPE film preparation avoids waste production (principle 1), saves atoms (principle 2), promotes energy efficiency (principle 6), uses renewable raw materials (principle 7), and is safe (principle 12), and the film is rapidly degraded in the environment (principle 10).

## Supplementary Material



## Notes

Grape pomace fractions’ appearance and its particle size distribution (PDF)
