# Supplementary material for: Optimizing Extraction of Bioactive Compounds from Grape Pomace to Produce an Eco-Friendly Smart Straw
Source: ACS Omega. 2026 Oct 15;11(42):64941–55. doi: 10.1021/acsomega.6c05389 (PMC13717778; doi:10.1021/acsomega.6c05389)

Supplementary material for the article: Optimizing extraction of bioactive compounds from grape pomace to produce an eco-friendly smart straw

Luis Fernando Zitei-Baptista[a], Maisa Mitiko Takahashi[a], Ivone Yanira Choquetico Iquiapaza[a], Delia Rita Tapia-Blácido[a*]

[a] Department of Chemistry, Faculty of Philosophy, Sciences and Letters of Ribeirão Preto, Ribeirão Preto, São Paulo, Brasil, 14040-901.

*Corresponding author: Delia Rita Tapia-Blácido (delia@ffclrp.usp.br), Department of Chemistry, Faculty of Philosophy, Sciences and Letters of Ribeirão Preto, Laboratory of Agroindustrial Biopolymers, Ribeirão Preto, São Paulo, Brasil, 14040-901.

Figure S1. Grape Pomace fractions' appearance and its particle size distribution

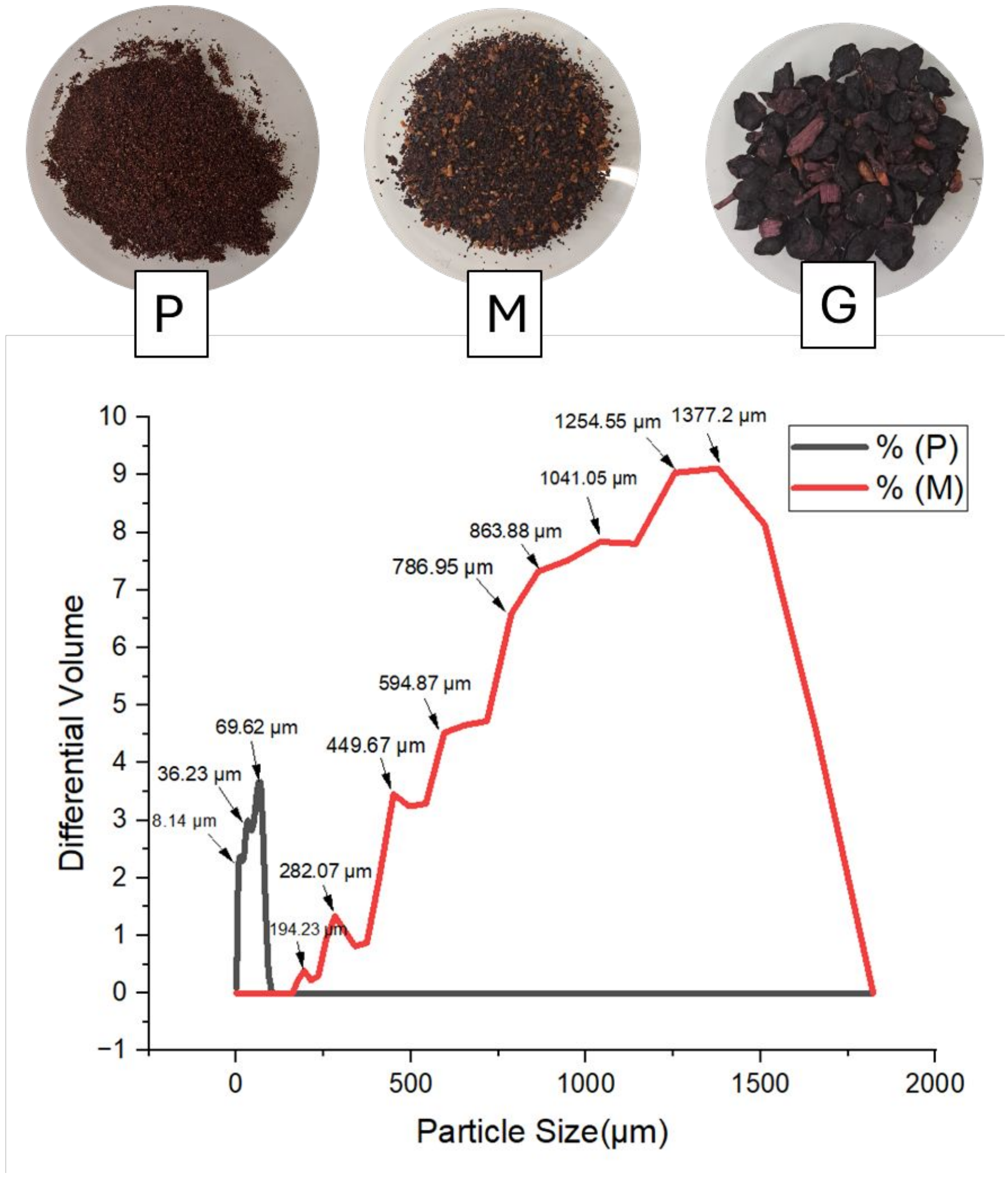

Supplement: Supplementary file 1 [file ao6c05389_si_001.pdf]
